# Balancing Maternal Melanoma and Vaginal Birth After Cesarean (VBAC) Delivery: A Case Report Highlighting Shared Decision-Making

**DOI:** 10.7759/cureus.66279

**Published:** 2024-08-06

**Authors:** Gregory Vurture, Brittany Klein, Richard Shapiro, Jonathan Baum

**Affiliations:** 1 Obstetrics and Gynecology, Hackensack Meridian, Jersey Shore University Medical Center, Neptune, USA; 2 Surgical Oncology, New York University Langone Medical Center, New York, USA

**Keywords:** malignancy, induction of labor, melanoma, vaginal birth after cesarean, trial of labor after cesarean

## Abstract

Melanoma is increasingly common among reproductive-age women and is one of the most common cancers diagnosed during pregnancy. The literature for melanoma in pregnancy, especially among those with prior uterine scars, is limited. We present an interesting case of a 22-year-old woman who went to her dermatologist for a suspicious lesion on her thigh. The lesion was excised, and histopathology confirmed that it was a melanoma. The dermatologist recommended immediate delivery. The patient then urged her obstetrician to undergo the risks of an induction of labor (IOL) for a trial of labor after cesarean (TOLAC) because she desired a large family, and a second cesarean would make this more difficult to happen. She ultimately had a successful vaginal birth after cesarean (VBAC) and subsequent excision of the melanoma with surgical oncology in the immediate postpartum period. Therefore, the decision for IOL for TOLAC in this case was based on the patient’s fears regarding melanoma disease progression in her 39th week. Given the short time course between delivery and excision of her melanoma, it is possible that she may have been able to wait for spontaneous labor, which would have avoided the risks associated with IOL for TOLAC. The optimal timing of surgical intervention for melanoma in pregnant patients who desire TOLAC is unknown. In pregnancies that are approaching their due date, waiting for spontaneous labor may be a reasonable approach to avoid the risks of labor induction, especially in women with prior cesarean delivery. A multidisciplinary approach involving dermatology, surgical oncology, and the obstetric team is warranted to optimize both dermatologic and obstetric outcomes.

## Introduction

Melanoma is increasingly common among reproductive-age women. It is one of the most common cancers reported in pregnancy, with an incidence of 1-3 out of 10,000 pregnancies [1]. In the United States, the incidence of melanoma varies based on ethnicity [2]. Non-Hispanic patients were most likely to have the disease [2]. The literature regarding the management of melanoma in pregnancy is limited. The decision to intervene becomes more complicated for those with a prior cesarean delivery. There is significant evidence to suggest that those undergoing an induction of labor (IOL) for a trial of labor after cesarean (TOLAC) are less likely to achieve a successful vaginal birth after cesarean (VBAC) compared to those who wait for spontaneous labor [3-6]. There is no data to guide delivery route decisions in pregnant patients with melanoma and a prior cesarean delivery. We present an interesting case of a patient in her third trimester who was found to have a spitz melanoma. She chose IOL for TOLAC to expedite surgical staging of the melanoma and to avoid another cesarean delivery.

## Case presentation

**Figure 1:**
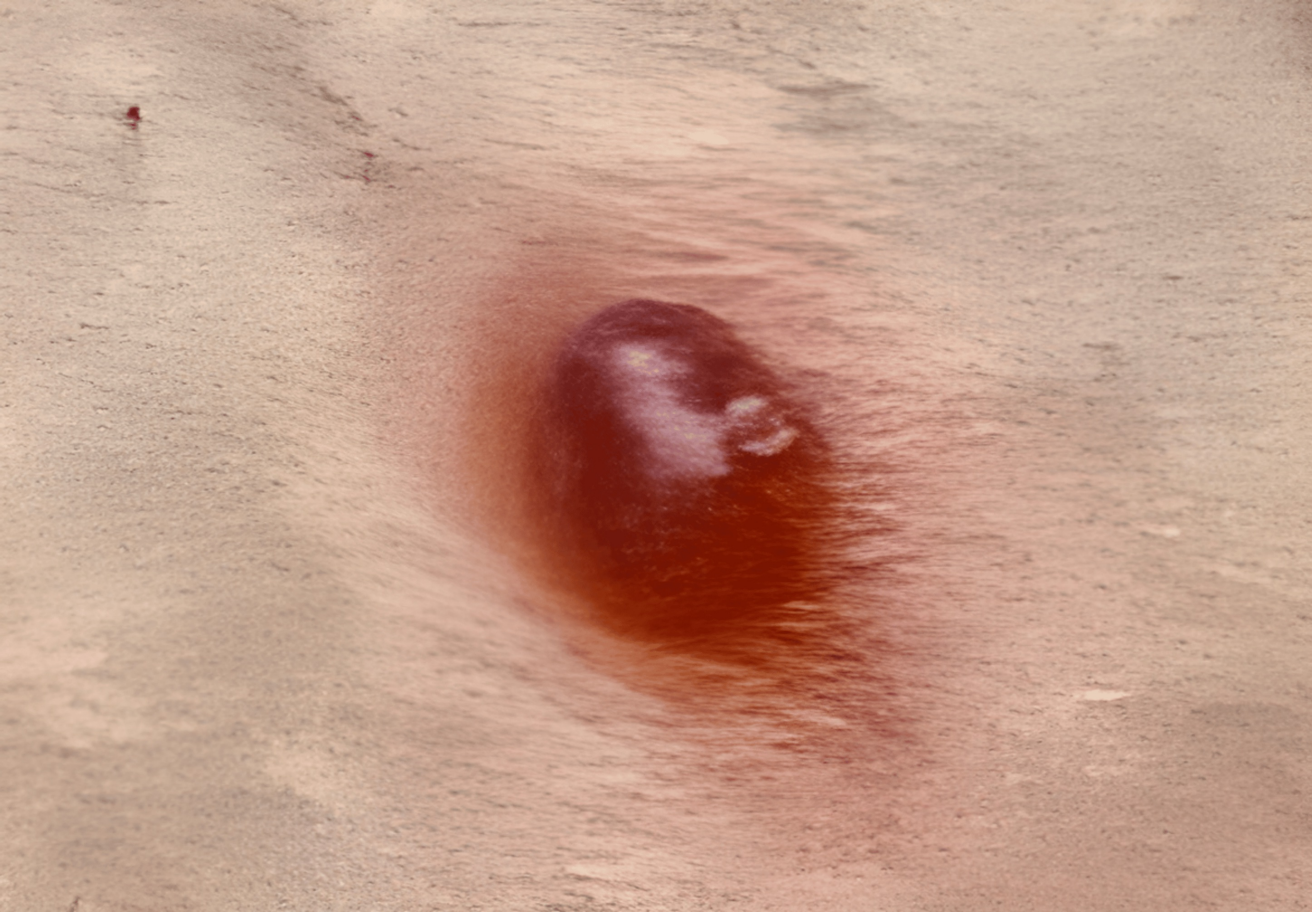
Dome shaped mole of left anterior thigh

A 22-year-old para 1 presented to the dermatologist at 35 weeks gestational age with a dome-shaped mole on her left anterior thigh (Figure 1). Obstetric history is significant for a prior cesarean delivery for breech presentation, with a strong desire for a large family and to avoid another cesarean.

**Figure 2:**
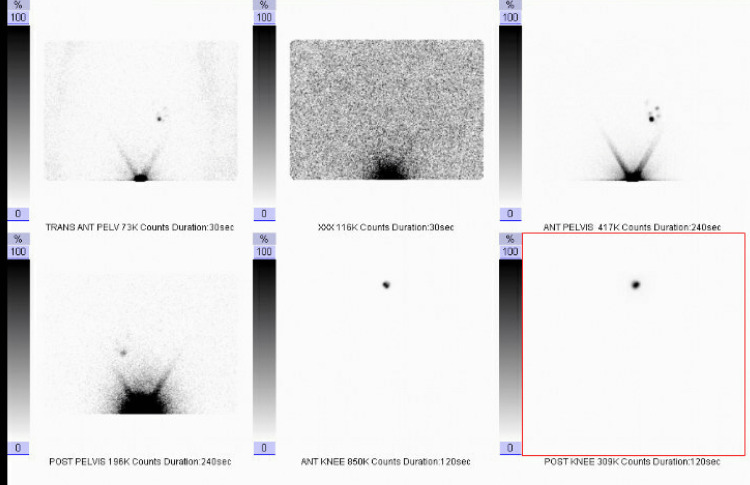
Lymphoscintigraphy demonstrating uptake of the left inguinal lymph node

The mole increased in size during her pregnancy. The final pathology resulted at 39 weeks and demonstrated spitz melanoma measuring 1.4 mm in Breslow thickness (Figure 2). BRAF status was negative. Dermatology recommended immediate delivery before receiving surgical staging and treatment for the melanoma.

The patient urgently reached out to her obstetrician to arrange for delivery. She wanted to avoid another cesarean unless necessary. She requested an IOL, which required the use of oxytocin. The patient went on to deliver a healthy male infant, Apgar scores 9 and 9, weighing 3.3 kg. Her induction, delivery, and postpartum courses were uncomplicated. The patient and newborn were discharged home within 24 hours.

**Figure 3:**
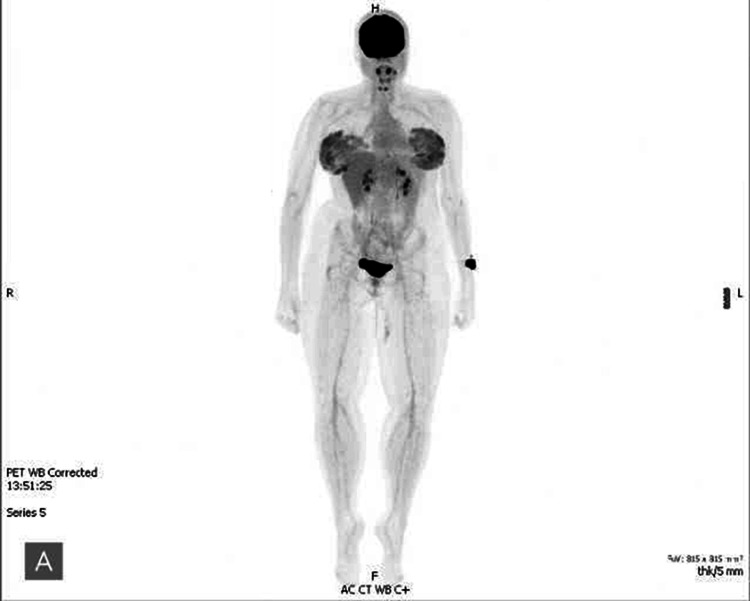
Positron emission tomography (PET) scan demonstrating no evidence of metastasis

Following delivery, surgical staging was scheduled. Preoperative lymphoscintigraphy was accomplished using filtered technetium radiolabeled sulfur colloid (Figure 3). She then underwent a wide and deep excision of the lesion with a sentinel lymph node biopsy. The surgical defect measured 15x5cm. A complex, layered wound closure was performed. The patient tolerated the procedure well without complications and was discharged the same day. 

The final pathology demonstrated pT2a spitzoid melanoma without ulceration or mitoses with scattered spitzoid cells in the sentinel lymph node biopsy of the left inguinal lymph node. The pathologic stage of the melanoma was reported to be Stage IIIA (pT2a, pN1a, cM0). Placental pathology showed no evidence of melanoma infiltration. Subsequent positron emission tomography (PET) scans demonstrated no evidence of recurrent local or distant metastasis. She was ultimately taken under surveillance with a physical exam and ultrasonography of the nodal basin every four months.

In regard to her gynecological health, she chose a levonorgestrel-intrauterine device (LNG-IUD) 52 mg as a long-term, reliable, and reversible contraceptive method while undergoing surveillance of her disease.

## Discussion

Melanoma is the one of the most common cancers diagnosed in pregnancy [1,7]. Benign moles may change during pregnancy, which can raise concern and complicate the diagnosis [7]. Often, lesions are classified as atypical Spitz nevi or indeterminate lesions. Nonetheless, a suspicious lesion in a pregnant woman warrants an immediate biopsy.

Currently, there is no concise recommendation regarding the optimal timing for surgical intervention for melanoma in pregnancy or postpartum. One may consider the gestational age to guide steps in management. In the first trimester, we typically begin the metastatic workup with ultrasonography for lymph nodes, followed by excision under local anesthesia. A wide excision and sentinel lymph node biopsy can then be performed later in pregnancy if indicated. In the third trimester, the same workup can be done, followed by a definitive excision and a sentinel lymph node biopsy postpartum. In the second trimester, the metastatic workup is performed with a sentinel lymph node biopsy and fetal monitoring after approximately 26 weeks of gestation; otherwise, preoperative and postoperative fetal heart sounds can be obtained. The timing of a sentinel lymph node biopsy in pregnancy is controversial. Some argue to wait for the postpartum period regardless of gestational age [8], while others recommend it be performed in the second trimester [9].

The patient in the present report did not require oncologic treatment; however, when oncologic treatment is needed, it must be individualized for the patient. Certain combinations of BRAF, MEK, and checkpoint inhibitors can be teratogenic, resulting in severe fetal outcomes [10]. Depending on the BRAF plus MEK inhibitor, patients must avoid lactation or future pregnancy anywhere from 2 weeks to 4 months after the last dose of the specific systemic immunotherapy. Checkpoint inhibitors may require between 3-5 months after the last dose before lactation or future pregnancy may be safe. In the situation of more advanced melanoma, in which one of these immunotherapies may be required, the recommendation is to wait approximately 2-3 years [11].

TOLAC is associated with an increased risk of both catastrophic maternal and fetal complications [3]. These include uterine rupture, endometritis, postpartum hemorrhage, and hysterectomy. Neonatal complications include a low Apgar score, acidosis, and neurologic injury [3]. Melanoma metastasis to the placenta and fetus is an uncommon manifestation that is typically associated with more advanced metastatic disease [12]. Regarding prognosis, most melanoma recurrences occur within 3-5 years of the current pregnancy, and reliable birth control is therefore advised [13]. It is important to note that despite the risk of placental metastasis and the even more rare risk of fetal metastasis, the absolute risk of this complication is low, and therefore the diagnosis of melanoma alone is not an indication for a cesarean section [12]. However, shared decision-making is advised in specific patient populations, such as our patient who already had a uterine scar and ultimately desired a VBAC.

Waiting for spontaneous labor is a reasonable approach to maximizing VBAC success while minimizing intervention. Approximately 80% of patients prior to 41 weeks of gestational age will undergo spontaneous labor [14]. Regarding melanoma, the time course of the disease in pregnancy has been somewhat controversial; however, multiple cohort studies have suggested no significant difference in mortality in those diagnosed with melanoma both during and outside of pregnancy [15-17]. Spontaneous labor is associated with a higher rate of VBAC success [2-5] and is therefore preferred. IOL for TOLAC with oxytocin is not contraindicated and may be reasonable for certain patients but is associated with greater risk when compared to spontaneous labor [3-6] and has been associated with higher rates of uterine rupture [18-19].

## Conclusions

Our patient had to balance a new cancer diagnosis, reproductive wishes, and delivery route uncertainties, knowing that delays in staging and treatment may worsen her oncological prognosis. Another cesarean would significantly decrease her ability to pursue a future vaginal delivery, and any postoperative complication(s) could impact or delay staging and treatment. The decision for IOL in this case was based on dermatologic concern for delayed staging and treatment as well as the patient's fears regarding melanoma spread. The risks of inducing labor in a woman with a prior cesarean scar may have outweighed the benefit of expediting treatment. Nonetheless, our patient had a successful obstetric and dermatological outcome. The decision of whether to intervene before or after delivery should therefore be individualized. Ultimately, further research is needed to determine the optimal timing of surgical intervention for melanoma diagnosed during pregnancy, timing of delivery, and delivery route, especially in those with a prior cesarean delivery.
